# Flint on “Abnormal Condition of the Kidneys in Bright’s Disease”

**Published:** 1856-11

**Authors:** 


We would desire to call particular attention to the article of Dr. Austin Flint on the "Abnormal Condition of the Kidneys in Bright's Disease," and shall make no apology for thus occupying so much of our space, the importance and obscurity of the subject and the ability of the author, fully justifying it.

